# Beneficial Effects on Oxidative Stress and Human Health by Dietary Polyphenols

**DOI:** 10.3390/antiox13111314

**Published:** 2024-10-29

**Authors:** Sonila Alia, Alice Di Paolo, Valentina Membrino, Tiziana Di Crescenzo, Arianna Vignini

**Affiliations:** 1 Department of Clinical Sciences, Università Politecnica delle Marche, 60100 Ancona, Italy; s.alia@pm.univpm.it (S.A.); a.dipaolo@pm.univpm.it (A.D.P.); v.membrino@pm.univpm.it (V.M.); t.dicrescenzo@pm.univpm.it (T.D.C.); 2 Research Center of Health Education and Health Promotion, Università Politecnica delle Marche, 60100 Ancona, Italy

During the past few years, researchers have dedicated themselves to studying phytochemicals which make up the nutritional and non-nutritional bioactive compounds found in fruits, vegetables, cereals, and other plant foods [1]. These phytochemicals are produced by plants for their protection, and have been much studied because of their great medicinal and nutritional value. Interestingly, scientific research has been focused on polyphenol properties for several reasons and not only because of their antioxidant and anti-inflammatory properties. First of all, polyphenols possess interesting health benefits in terms of their potential to prevent non-communicable and lifestyle-related chronic diseases, such as cardiovascular disease, diabetes, osteoporosis and cancer [2,3]. Connected to this, a growing field in scientific research has revealed that polyphenols may affect the composition of the gut microbiota, with implications for metabolic and immune health [4]. In addition, the growing focus on healthy and sustainable diets has led to a greater consumption of fruits, vegetables and beverages such as tea and coffee, all high in polyphenols. This has encouraged research into their beneficial properties and into how their role in plant growth and resistance can contribute to more sustainable agricultural practices [5,6]. Due to the advances in analytical technologies, it has become easier to isolate and study polyphenols, facilitating the understanding of their mechanisms of action and interactions with an organism [7]; in addition, the growing interest in and awareness of nutraceuticals, i.e., foods that provide therapeutic benefits, has prompted research into how polyphenols can be used in supplements or as components of a diet to improve health [8].

These factors, together with a growing interest in nutrition and public health, have made the study of polyphenols a dynamic and vibrant area of scientific research.

In the light of these considerations, we have put together this Special Issue, titled “Beneficial Effects on Oxidative Stress and Human Health by Dietary Polyphenols”. Although we primarily focus on cacao flavonoids, bioactive compounds with potential benefits in the prevention of chronic diseases associated with inflammation, oxidative stress (OS) and metabolic disorders, we intended to consider the general properties of polyphenols and aimed at opening new avenues in the treatment against oxidative damage. Here, we offer an overview of the content of this Special Issue, which comprises three original articles and one systematic review.

Great emphasis has been placed on waste generated from food processing to the point that the decrease in and valorization of agri-food byproducts is one of the Sustainable Development Goals established by the Agenda 2030 [9]. Cocoa bean shells (CBSs) represent the outer part of the cocoa bean and accounts for between 10 and 20% of the total cocoa bean weight. It is rich in carbohydrates, dietary fibers, fats, phenolic compounds, antioxidants and vitamins. CBSs are normally considered waste derived from the chocolate industry and are thrown away or used in low-value applications (animal feed or fertilizer). But in the last few years, the exploitation of CBSs is gaining attention for its potential as a useful and sustainable coproduct in the food industry and beyond [10]. Some of the main opportunities for exploitation are related to their use in food preparation as a nutraceutical ingredient employed in nutrition supplements; in the cosmetics industry; for the production of biomaterials such as bioplastics or building materials, helping to reduce environmental impact; or to produce renewable energy, thus contributing to sustainable waste management. In the manuscript by Rossin, D., et al., the authors conducted an in vitro study aimed at describing the positive effect of CBSs against oxysterol-induced oxidative and inflammatory intestinal barrier damage [11]. Oxysterols are the oxidative cholesterol derivatives, which are involved in inflammation, fibrosis, and cell death; they are also reported to play a role in the pathogenesis of several chronic diseases related to altered intestinal permeability [12]. The authors reported beneficial effects of CBSs and CBS-enriched ice cream against the disrupting action of an oxysterol mixture on intestinal cell monolayer in terms of reduced inflammation and OS. These positive effects are suggested to be due to the richness of CBS in polyphenols and bioactive compounds, making this byproduct an interesting supplement as a food ingredient, aiming to achieve potential benefits to health.

In line with this study, other authors developed a potential functional food by combining cocoa powder and carob flour in order to obtain a cocoa–carob blend (CCB) to be used as a prebiotic for improving intestinal dysbiosis and producing bioactive phenolic compounds derived from gut microbiota [13]. To assess this hypothesis, authors tested the effects of a 12-week supplementation with a CCB on the intestinal health of an animal model of type 2 diabetes (T2D), i.e., Zucker diabetic fatty (ZDF) rats. They used a model of T2D because during the past few years, the role of the intestine and the ecosystem of microbes living there in the development and progression of the disease has been increasingly documented [14]. García-Díez, E., et al. [13] showed for the first time that the consumption of a CCBs, which are rich in polyphenols, is able to prevent intestinal inflammation and OS in order to improve the gut barrier integrity, and in addition, to modify the composition of the intestinal microbiota, stimulating the growth of “good” bacteria and inhibiting the “bad” ones. Thus, CCB could be a useful prebiotic to support gut microbiota and in turn reduce diabetic complications. Related to these aspects, another therapeutic target in the management of diabetes, is insulin resistance (IR), which represents a major cardiovascular risk factor. In their research, Sani, L., et al. [15] examined a standardized dose of poplar propolis extract powder (PPEP) in non-diabetic insulin-resistant volunteers with obesity in order to verify its effect on glucose homeostasis and insulin resistance indicators. Bee propolis is a natural resinous mixture rich in more than three hundred chemical components, including polyphenols, steroids, vitamins and pollens, with documented biological activities [16]. In many animal studies, propolis has evidenced its efficacy on glucose and lipid metabolism and for antioxidant properties [17]. Three months of supplementation with 6 mg of total polyphenols/kg of body weight as part of a standardized PPEP resulted in the regulation of insulin homeostasis, thus preventing the development of diabetes when insulin resistance was present. A key contributing factor of diabetes is OS caused by the excessive production of pro-oxidants relative to antioxidant defenses. Interestingly, increased levels of reactive oxygen species (ROS) stimulate bone tissue degradation by inhibiting osteoblastogenesis and inducing osteoclastic differentiation [18]. In the last few years, emerging research has demonstrated the positive effect of polyphenols on osteoporosis by inhibiting the expression of markers involved in bone resorption. Using this idea as a starting point, Salvio, G., et al. [19] wrote a systematic review and meta-analysis on the effects of polyphenols on bone mineral density (BMD) and the markers of bone turnover (BTMs) in postmenopausal women. The authors studied twenty-one humans through randomized controlled trials (RCTs), all with parallel design except for one that had a crossover design. Studies comprised 3–36 months of supplementation with polyphenols, a separate evaluation of BMD at different sites (lumbar, femoral neck, hip, and whole-body) and the consideration of the main BTMs, such as serum bone-specific alkaline phosphatase (BALP), urinary deoxypyridinoline (DPD), serum osteocalcin (OC) and urinary pyridinoline (PD). In these studies, many took into consideration the effects of soy isoflavones in the prevention of bone loss. Despite the encouraging results, existing clinical evidence does not support the epidemiological data on decreased fracture risk related to a polyphenol-rich diet; in addition, the authors concluded that possible gastrointestinal side effects limited their beneficial potential consumption as supplements in postmenopausal women.

In conclusion, the scientific publications collected in this Special Issue highlight different roles played by polyphenols, and the by-products of the food industry that are rich in polyphenols, on reducing the occurrence of chronic diseases. Numerous mechanisms of action have been proposed to explain these protective properties. However, additional human studies are required to establish definite evidence concerning the favorable effects of polyphenols, since the number of clinical trials published so far remains limited.

While acknowledging the limited scope of this collection, the guest editors hope that these works will contribute to the field in the future, and it is expected that both the general population and individuals at risk of specific diseases will benefit the most from the effective use of polyphenols and optimal levels of consumption. This topic is also a fundamental concern in regard to dietary supplements, since their use can lead to a significant increase in polyphenol consumption, frequently beyond the levels naturally obtained from regular nutrition. All these notions are at the basis of personalized nutrition, a pioneering approach that could modernize the way we think about food and health. Once we are able to adapt our nutritional recommendations to genetic, metabolic and microbiological profiles, we will optimize the benefits of phytonutrients found in plant foods.
